# Moxifloxacin Concentration and Proteomic Analysis of Aqueous Humor in Human Uveitis Associated with Oral Moxifloxacin Therapy

**DOI:** 10.2174/1874364101711010107

**Published:** 2017-06-12

**Authors:** David M Hinkle, Nicole A Kruh-Garcia, Jonathan N Kruh, Carolyn Broccardo, Priyanka Doctor, C Stephen Foster

**Affiliations:** 1 Department of Ophthalmology, University of Massachusetts Medical School, Worcester, Massachusetts, USA; 2 Department of Microbiology, Immunology and Pathology, Colorado State University, Fort Collins, Colorado, USA,; 3 Jamaica Hospital Medical Center, Queens, New York, USA; 4 Research Integrity and Compliance Review Office, Colorado State University, Colorado, USA; 5 Massachusetts Eye Research and Surgery Institution, Cambridge, Massachusetts, USA; 6 Ocular Inflammation and Uveitis Foundation, Cambridge, Massachusetts, USA; 7 Harvard Medical School, Boston, Massachusetts, USA

## Abstract

**Purpose:**

The aim was to report the aqueous humor moxifloxacin concentration and proteome profile of an individual with bilateral uveitis-like syndrome with pigment dispersion.

**Methods:**

Multiple reactions monitoring mass spectrometry quantified the aqueous concentration of moxifloxacin in the affected individual. Shotgun proteomic analysis performed via liquid chromatography tandem mass spectrometry (LC-MS/MS) defined the protein profile in the affected individual and unaffected control samples.

**Results:**

Moxifloxacin was present at higher than expected levels in aqueous humor 18 days following oral administration. One-third of the proteins were identified by significantly lower spectral counts in the aqueous of the individual with moxifloxacin associated uveitis compared to the unaffected control.

**Conclusion:**

Moxifloxacin was detected in aqueous humor 18 days following the completion of oral administration. These results suggest that moxifloxacin toxicity may be responsible for the uveitis-like syndrome with pigment dispersion syndrome induced by moxifloxacin therapy.

## INTRODUCTION

Fluoroquinolones are a broad spectrum antibiotic class indicated for the treatment of bacterial infections including acute exacerbation of chronic bronchitis, complicated intra-abdominal abscess, community acquired pneumonia, acute sinusitis and uncomplicated skin infections. Fluoroquinolone antibiotics inhibit DNA gyrase activity, thereby disrupting bacterial DNA replication and transcription. Adverse drug reactions unique to quinolones include tendinitis, tendon rupture, peripheral neuropathy and diplopia. Uveitis was not reported in premarketing trials in the package inserts. Following the initial report of bilateral panuveitis associated with oral moxifloxacin therapy in 2004, there have been additional reports of a bilateral uveitis-like syndrome with anterior chamber pigment dispersion and iris transillumination [1-3].

We report the moxifloxacin concentration and proteomic analysis of aqueous humor obtained during the acute phase of bilateral pigment dispersion with elevated intraocular pressure and subsequent development of diffuse iris transillumination.

The goals of this study were to establish if moxifloxacin is sequestered in aqueous humor following systemic administration and to determine whether the aqueous proteome is altered by the condition. While this phenomenon has been described clinically, the full characterization of aqueous humor to corroborate the observation has not been published. A two-pronged approach using mass spectrometry was employed to perform both relative quantification of moxifloxacin, and protein content within aqueous of affected and control eyes.

## METHODS

A 71 year old Caucasian female developed bilateral painful red eyes with photophobia 14 days following inpatient treatment for pneumonia with moxifloxacin (Bayer HealthCare Pharmaceuticals, San Francisco, CA) 400mg daily. There was no past history of uveitis, glaucoma or pigment dispersion. Best corrected visual acuity was 20/200 bilaterally (LogMAR 1.00). Microcystic corneal edema secondary to elevation of intraocular pressure greater than 40mmHg in both eyes developed despite maximal topical glaucoma therapy and oral acetazolamide 500 mg twice daily. Moxifloxacin associated uveitis was suspected due to bilateral, extensive pigment dispersion present in the anterior chamber. Anterior chamber paracentesis for diagnostic and therapeutic purposes was performed 18 days following the last oral dose of moxifloxacin. A peripheral corneal paracentesis incision was made with a sterile 15 degree steel blade and 0.15 mL of aqueous fluid was harvested from the right eye (OD) and 0.27 mL was harvested from the left eye (OS) using a blunt 27 gauge cannula. Aqueous humor samples were stored frozen at -80 °C and transported on dry ice for quantification of moxifloxacin concentration and proteome analysis.

Urgent bilateral glaucoma drainage device surgery was required due to intraocular pressure elevation greater than 40mmHg despite maximal medical therapy. Pigment dispersion persisted for more than one year with subsequent development of bilateral iris transillumination and cataracts in both eyes. Bilateral posterior synechiae were observed at the time of cataract surgery and the early post-operative course was complicated by formation of fibrin in the anterior chamber.

One unaffected patient consented to harvest of aqueous humor via corneal paracentesis immediately prior to cataract surgery (control). Written informed consent was obtained from each subject. This study was performed in accordance with the Declaration of Helsinki.

Mass Spectrometry Sample Preparation: Moxifloxacin quantification was performed for each of the three samples (OS, OD and control). Proteins were precipitated using 4 volumes of cold (-20 °C) methanol. Starting volumes isolated from patients were: 150 µL control, 150 µL OD and 270 µL OS. Samples were incubated for 30 min at -80 °C and centrifuged for 10 min at 10000 rpm at 4 °C. Supernatants were dried and re-suspended in TQ-S Buffer A (0.1% formic acid in water) for multiple reaction monitoring (MRM) mass spectrometry analysis. The protein pellets were then analyzed with LTQ shotgun proteomic analysis.

Protein pellets were re-suspended in 10 mM ammonium bicarbonate (ambic) and quantified by micro bicinchoninic acid assay (mBCA). The total proteins in the samples are as follows: Control (11.3 µg), OD (32.8 µg) and OS (177.7 µg). To normalize the protein samples, only 10 µg were used in the protein analysis. Samples were digested with trypsin via in-solution protocol as previously described [4]. Briefly, samples were denatured in 6 M guanidine hydrochloride, reduced with 10 mM DTT and alkylated with 100 mM iodoacetamide. After overnight micro-dialysis in 10 mM ammonium bicarbonate, the samples were digested with trypsin (1:50, trypsin:sample) in 10% acetonitrile (ACN) overnight at 37 °C. Digests were dried and re-suspended in LTQ loading buffer (3% ACN and 0.1% formic acid, in water). All digests were performed in de-plasticized tubes to reduce plastic polymer contamination.

Relative Quantification of Moxifloxacin by Multiple Reaction Monitoring (MRM) Mass Spectrometry: All the analyses were performed on an LC-MS/MS system consisting of Waters nanoACQUITY UPLC coupled to a Waters Xevo TQ-S mass spectrometer fitted with TRIZAIC source. The instrument was operated in positive electrospray ionization mode using MassLynx V4.1 SCN810 (Waters, Milford, MA). Chromatography was performed on 150 μm × 50 mm TRIZAIC™ nanoTile packed with BEH C18 1.7 μm. Injections were 2 µl using partial loop mode. The initial solvent composition was 90% A (0.1% formic acid in water) and 10% B (0.1% formic acid in methanol). The mobile phase gradient was: 10-55% B from 0.25-1 min, 55-95% B from 1-15.5 min, 95-10% B from 15.5-16 min, and a hold at 10% B from 16-20 minutes. The flow rate was 3.06 µL/min and the column was maintained at 46 ^o^C. The cone voltage was static at 50 V. The capillary voltage was 3.2 kV, source temperature was 100 ^o^C, source offset was 50 V, and the collision gas was argon. Dwell time for all compounds was 0.830 s. Moxifloxacin parent mass to charge ratio (m/z) was determined to be 401.175. The two daughter ions monitored were m/z 358 and m/z 384, corresponding to the loss of CO_2_ and H_2_0 respectively [5]. Collision energy necessary to generate the daughter species was determined to be 20 V (for the loss of CO_2_) and 30 V (for the loss of H_2_0). Patient samples were randomized and injected in triplicate.

MRM Data Analysis: Quantification of patient samples was done using linear regression against a standard curve in TargetLynx^TM^ (Waters, Milford, MA). Curves were generated from the ophthalmic solution moxifloxacin in 0.5% HCI (Vigamox, Alcon, Fort Worth TX) utilizing the control patient sample as a representative background matrix. Eight concentrations ranged from 0.1 – 1000 nmol/ µL, and each sample was injected in triplicate from which one regression equation was generated per ion Fig. (**1**). The concentration of each daughter ion in Control, OD and OS sample was calculated based on this data (Table **1**). Peak identification was performed using MassLynx software version 4.1. Upon examination and standardization of the peaks, the transition results were exported to Excel^TM^ (Microsoft, Redmond WA) for calculation of calibration curves (R^2^ values for each curve are shown in (Table **1**), as well as determination of limits of detection (LOD) and quantitation (LOQ) for each transition [6, 7]. LOD = 3.3 (SD/S) and LOQ = 10 (SD/S), where SD is the standard deviation of y values and S is the slope of the calibration curve. SD of y was calculated in Excel^TM^ using the LINEST function (Table **2**).

Mass Spectrometry of tryptic peptides. LC-MS/MS methods for proteomic analysis were previously published [7]. All samples were injected at a concentration of approximately 500 ng/µL. Peptides were purified and concentrated using an on-line enrichment column (Agilent Zorbax C18, 5 µm, 5×0.3 µm column, Agilent 1100 nanoHPLC,). Subsequent chromatographic separation was performed on a reverse phase nanospray column (Zorbax C18, 5 µm, 75 µm ID × 150 mm column). Samples were eluted into an LTQ linear ion trap (Thermo Scientific, Waltham, MA) using a flow rate of 300 nL/min with a linear gradient of acetonitrile. Mass spectra were recorded over a m/z range of 200–2000 Da using a dynamic exclusion limit of 2 MS/MS spectra of a given mass for 30 s (exclusion duration of 90 s). Compound lists of resulting spectra were generated using Bioworks^TM^ 3.0 software (Thermo Scientific) with intensity threshold of 5,000 and 1 scan/group. All the samples were run in triplicate.

Database searching and Criteria for Protein Identification. All tandem mass spectra (.raw files) were extracted by LCQ_DTA.exe (Thermo Scientific) for subsequent loading into the Mascot (Matrix Science, London, UK; version: 2.3.02 Mascot MS/MS search engine or into Bioworks Browser (version 3.3.1 SP1) for subsequent analysis with Sequest (Thermo Finnigan, San Jose, CA; version SRF v.27, rev. 11) and X! Tandem (version CYCLONE 2010.12.01.1) [8]. All three search engines were used to query the Universal Protein Resource (UniProt) human database (created on 11/26/12) composed of 169776 entries including reverse proteins for false discovery calculation. All searches were performed assuming trypsin digestion, with a fragment ion mass tolerance of 1.5 Da, a parent ion tolerance of 2.5 Da. Oxidation of methionines (+16) and iodoacetamide derivative of cysteine residues (+57) were specified as variable modifications.

 All data was compiled using Scaffold (version 3.6.1, Proteome Software Inc., Portland, OR) in order to validate MS/MS based peptide and protein identification. Peptide identifications were accepted if established using the following scores: Mascot: Ion Identity: 0, Ion score +1, +2 and +3 all at 55; Sequest: DeltaCn: 0.06, XCorr +1, +2 and +3 at 1.05, 2.1 and 3.15, respectively; X! Tandem: -Log(E-Value): 2. Protein identifications were only accepted if established at greater than 99% probability and contained at least two identified peptides. Protein probabilities were assigned by the Protein Prophet algorithm [9]. False discovery rate (FDR) for proteins was calculated to be 3% at the protein level and 0.1% at the peptide level (Table **3**). Raw and searched data files are accessible via http://proteomecentral.proteomexchange.org/cgi/GetDataset.

## RESULTS

MRM method development on ophthalmic moxifloxacin solution afforded monitoring of the parent compound (m/z 401.175) and upon fragmentation, two daughter species, m/z 358 and m/z 384, corresponding to loss of CO_2_ and H_2_0 respectively. Relative quantification of clinical samples was based on dosage response curves generated from this solution Figs. (**1** and **2**).

 As expected, the control sample did not contain detectable levels of moxifloxacin. The transition ion m/z 358 was detected well above both the LOD and LOQ in aqueous humor samples from both affected eyes. This data was used to quantitate the amount of moxifloxacin in these samples. The transition ion m/z 384 was below the LOQ and could not be used to quantitate the amount of moxifloxacin, however, since the concentration was above that of the LOD we can use it as a confirmatory ion, enhancing the confidence of the detection. The levels determined by this technique, while not absolute, are consistent with the level of moxifloxacin calculated to be present roughly 2.5 days post administration, rather than 18 days, based on the previously reported intraocular concentration and pharmacokinetics of orally administered moxifloxacin.

The protein fraction of aqueous was analyzed by shotgun tandem mass spectrometry. Thirty-three proteins were confidently in aqueous of affected eyes and 32 proteins in an unaffected control eye. Ten proteins were significantly reduced in affected eyes compared to control (Table **4**).

## DISCUSSION

More than 50 cases of bilateral simultaneous uveitis-like syndrome with pigment dispersion and iris transillumination have been reported following systemic administration of moxifloxacin or levofloxacin [1, 2, 10]. Because iris transillumination is frequently observed in the light colored irides of patients with herpetic uveitis, some patients with uveitis following oral fluoroquinolone therapy were treated with oral anti-viral therapy without favorable effect on the course of the condition [10]. Furthermore, qualitative polymerase chain reaction analysis of aqueous humor obtained from 4 of 5 patients did not demonstrate amplification of DNA for herpes simplex, herpes zoster, cytomegalovirus or Epstein-Barr virus genomes [2, 10]. One individual with prior history of uveitis had PCR evidence of HSV viral genome in aqueous [2]. To our knowledge, none of the previously reported individuals were tested for, nor exhibited findings typical of, human herpes virus 6 (HHV6) associated uveitis, i.e. panuveitis in association with optic papillitis; however, it appears unlikely that a virus in the herpes family is the causative agent for the condition based on disparity between the clinical signs and lack of therapeutic response to empiric antiviral therapy.

Large, retrospective cohort studies have yielded conflicting results regarding the risk of uveitis and the use of oral fluoroquinolones. In one cohort, oral administration of fluoroquinolones confered an adjusted relative risk of 3.53 for development of uveitis within the subsequent 30 days [11]. The relative risk of uveitis was also increased in patients receiving oral macrolide and beta lactam antibiotics. The authors postulated systemic infectious disease, rather than the antibiotic administered, might be the cause of uveitis observed in these cases [11]. A second case-control study reported similar adjusted rate ratios of 2.98, 1.96 and 1.26 for the development of uveitis in first time users of moxifloxacin, ciprofloxacin and levofloxacin, respectively, compared to age and gender matched controls [12]. A third study calculated the hazard of uveitis development was not elevated after a fluoroquinolone prescription compared with the hazard of uveitis following a beta-lactam prescription and identified the risk for uveitis-associated systemic illnesses as a potential source of bias in the previous studies [13].

**Fig. (2):**
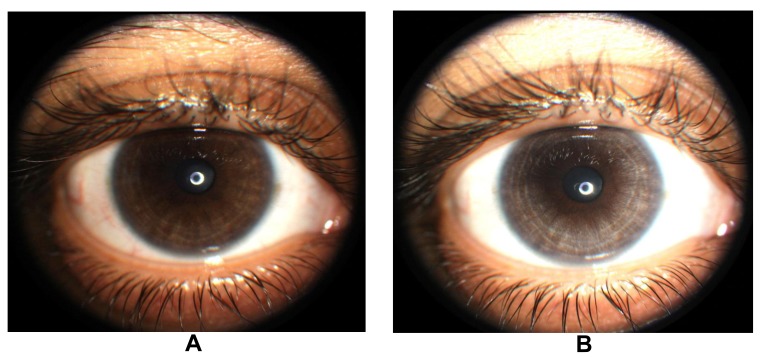
Slit lamp photomicrograph demonstrating progressive depigmentation of the iris stroma of the right eye (Panel A photo taken in 2012 and Panel B taken in 2013) in an individual following topical administration of moxifloxacin.

Tugal-Tutkin termed the condition bilateral acute iris transillumination (BAIT) and postulates it may be caused by an emerging respiratory pathogen rather than an adverse drug reaction because antibiotic therapy was not known to precede the condition in 38% of 26 cases [14]. Prior moxifloxacin therapy was identified in 35% of cases and antibiotics other than flouroquinolones were identified in 27% of cases. Tugal-Tutkin also described bilateral acute depigmentation of the iris (BADI), in contrast to BAIT which preferentially affects the iris pigment epithelium, a condition with predilection for loss of iris stromal pigmentation which is reversible in some cases [15]. BAIT may be a severe, irreversible form of BADI. Unilateral depigmentation of the iris was observed by one the authors (PD) following ipsilateral topical moxifloxacin therapy in an individual from India suggesting that local moxifloxacin therapy may rarely produce similar ocular findings (Fig. **2A**, **B**).

Orally administered moxifloxacin crosses the blood ocular barrier of non-inflamed human eyes and achieves concentrations of 1.58 +/-0.80µg/mL within 4 hours of administration of a single 800mg dose [16]. Rabbit studies of intravitreal moxifloxacin clearance report a vitreous half-life of 1.72 hours [17]. Because the serum half-life of orally administered moxifloxacin is 12 hours, moxifloxacin should be undetectable in human serum 5 days following oral administration [18]. Assuming human pharmacokinetics is comparable to the rabbit model, moxifloxacin should be undetectable in vitreous humor within 12 hours of a single intravitreal injection. Although aqueous clearance of antibiotics and other pharmaceuticals is typically more rapid than vitreous clearance in animals and humans, vitreous clearance of moxifloxacin may be slower in humans than animals. If we assume elimination of moxifloxacin from human aqueous is equivalent to first order elimination of moxifloxacin from rabbit vitreous then the concentration of moxifloxacin present in aqueous on the last day of systemic administration can be calculated as 3023 grams using the formula:

Initial concentration = Final concentration / 2^n^ where n = half life

This amount vastly exceeds the total systemic dose (4 grams) the patient received. There are several possible explanations for persistent moxifloxacin detected in aqueous humor 18 days following oral administration. Antibiotic clearance is generally believed to be delayed in inflamed eyes. Supra-therapeutic levels might result despite following recommended dosing due to impaired drug metabolism and elimination with resultant toxicity and shedding of iris and ciliary body pigment epithelium. Affected individuals may have a heretofore unrecognized enzyme deficiency impairing moxifloxacin elimination. To our knowledge, only one case exhibited co-morbid systemic symptoms of moxifloxacin toxicity [10]. Alternatively, moxifloxacin clearance from the anterior chamber may be impaired by the tremendous amount of pigment dispersion blocking aqueous egress through the trabecular meshwork. Serum moxifloxacin concentration was not determined in our patient nor reported in any prior publications, thus it is not clear whether persistent elevation of moxifloxacin is a local or systemic phenomenon

Knape *et al.* observed only phakic individuals have been reported to develop BAIT and suggest that drug may be trapped behind the iris by posterior synechiae which they confirmed via optical coherence tomography in one patient [19]. Not all patients with the condition have posterior synechiae [10]. One would expect the pupil would need to be completely secluded in order to sequester drug or aqueous in the posterior chamber. Pupil seclusion should result in iris bombe and secondary angle closure but this has not been observed. Conversely, some patients have abnormally deep anterior chambers with reverse papillary block or iris concavity configuration similar to the typical pigment dispersion patient [3, 18].

Intraocular injection of moxifloxacin is advocated for the prophylaxis and treatment of post-operative endophthalmitis. There are no reports of toxicity with single dose intraocular use to date. Multiple intravitreal injections (mean number injections was 6) of moxifloxacin 165 µg/0.1mL combined with pegaptanib (Macugen, Valeant Pharmacueticals, Montreal, QC) every 6 weeks as treatment for wet macular degeneration was well tolerated in 80 eyes of 65 human patients during 13.2 months median follow-up [20].

Toxicity studies have not detected adverse effects during the 14 days following intravitreal injection of doses up to 160 µg /0.1mL; however, a dose of 320 µg/0.1mL led to marked decreases in ERG findings in rabbits [21]. Concentrations higher than 150 µg/mL have adverse effects on primary retinal pigment epithelial cells in culture and concentrations of 500 µg/mL led to occasional, isolated retinal necrosis in mice [22, 23].

Phototoxicity is a well-known class effect of fluoroquinolones which has been postulated to play a role in the pathogenesis of iris atrophy; unlike other fluoreoquinolones, phototoxicity is not a reported adverse effect of oral moxifloxacin [2, 24]. Phototoxicity does not explain the selective involvement of iris pigment epithelium. The ciliary body is also presumed to be involved given the loss of accommodation experienced by young individuals with BAIT [10]. Retinal pigment epithelium does not demonstrate any abnormality on clinical examination. To our knowledge, retinal electrophysiologic studies have not been performed on any affected individuals. Dermal phototoxicity has not been reported as a feature of BAIT. The failure to determine serum moxifloxacin concentration at the time of aqueous harvest is a weakness of this study.

Because the few PCR studies performed in patients previously afflicted with this condition were unrevealing, we performed proteome analysis in an effort to further elucidate the pathogenesis of the condition [2, 10]. Interpretation of results of proteomic analysis of the human eye in normal and disease states is limited by our incomplete, albeit rapidly expanding, knowledge. The aqueous humor proteome of otherwise healthy patients undergoing cataract surgery was initially reported to contain at least 54 unique proteins, comprised of albumin-bound, albumin-depleted fractions and 8 proteins common to both fractions [25]. A subsequent study of eyes undergoing cataract surgery identified 676 unique aqueous proteins [26]. Depletion of albumin, the dominant protein identified in these samples, as well as fractionation of the protein content of the aqueous humor would enhance the number of proteins identified in this experiment. The authors speculate that not only would additional proteins be revealed by such processes, but additional peptides would be identified—further increasing our confidence in the proteins identified. Due to the confounding level of albumin in this data set, it is unclear whether moxifloxacin present in the aqueous resulted in the low spectral counts for one-third of proteins identified. To our knowledge, there are not reports of aqueous proteome analysis in human idiopathic uveitis and the lack of a patient with idiopathic uveitis serving as a control is a weakness of this study.

A second limitation of proteome analysis methods is that interpretation of shotgun proteomic data is hampered by the so called ‘protein inference problem’; the same peptide sequence can be present in multiple different proteins or isoforms leading to ambiguities in determining protein identity [27]. Proteome analysis did not aid in determining the etiology of the condition but as methods continue to evolve, this technique may be a useful adjunct in ascertaining the etiology of idiopathic ocular inflammatory conditions including BADI and BAIT.

## Figures and Tables

**Fig. (1):**
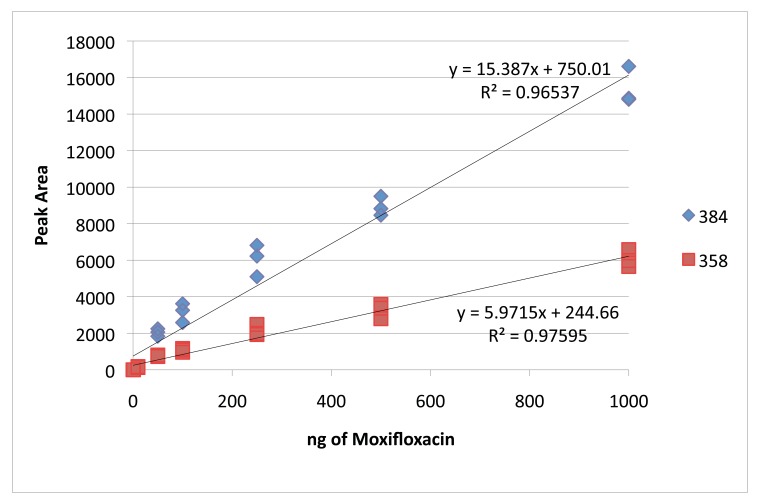
Calibration curve for both transitions of moxifloxacin determined from response in matrix. See (Table **1**) for results.

**Table 1:** Results of calibration curve calculation and determination of limits of detection and quantitation.

**Ion**	**R^2^**	**SD of y**	**LOD (ng)**	**LOQ (ng)**
358.00	0.98	51.42	25.83	86.11
384.00	0.97	63.29	12.34	41.13

**Table 2:** Concentration of moxifloxacin in the patient samples. The transition ion m/z 358 was well above both the LOD and LOQ in both of the samples and therefore was used to quantitate the amount of moxifloxacin in the original more dilute samples. The transition ion m/z 384 was below the LOQ and could not be used to quantitate the amount of moxifloxacin, however since the concentration was above that of the LOD we can use it as a confirmatory ion, enhancing the confidence of the detection.

**Sample**	**Ion**	**Role**	**calculated concentration injected (ng/µL)**	**concentration of moxifloxacin in sample (ng/µL)**
OD	358.00	Quantitation	268.32	44.72
OD	384.00	Confirmation	20.73	below LOQ
OS	358.00	Quantitation	101.33	93.82
OS	384.00	Confirmation	27.38	below LOQ

**Table 3:** List of proteins identified in the control and two patient samples. Three technical replicates were performed for each biological sample and the spectral counts determined for each is listed. A single protein which matched to the reverse database (in bold) is responsible for the FDR rate of 3%.

#	Identified Proteins (33)	**Samples and Technical Replicates**								
		**Control**			**OD**			**OS**		
		**1**	**2**	**3**	**1**	**2**	**3**	**1**	**2**	**3**
1	Serum albumin	1065	1008	1158	1157	1412	1049	1418	1253	1062
2	Serotransferrin	197	241	231	99	125	47	112	115	41
3	Alpha-1-antitrypsin	13	9	12	24	23	17	23	16	20
4	Ig gamma-1 chain C region	22	31	17	11	12	8	11	8	3
5	Prostaglandin-H2 D-isomerase	28	37	29	12	8	1	3	11	2
6	Transthyretin	15	16	12	24	25	7	20	13	7
7	Ig kappa chain C region	13	22	24	8	7	4	16	6	2
8	Alpha-1-acid glycoprotein 1	9	9	9	20	14	8	14	10	7
9	Hemopexin	7	7	7	22	18	8	9	6	1
10	Ig lambda-2 chain C regions	15	17	13	12	8	1	15	16	6
11	Complement component C4B	8	8	8	8	7	4	5	9	3
12	Pigment epithelium-derived factor	16	17	14	3	5	1	5	3	2
13	Vitamin D-binding protein	2	4	4	6	6	6	8	10	5
14	Ceruloplasmin	7	8	12	2	3	1	1	1	1
15	Alpha-1B-glycoprotein	1	7	8	5	4	3	5	9	6
16	Isoform 2 of Clusterin	14	5	4	8	7	2	4	6	3
17	Apolipoprotein A-I	3	0	5	8	3	4	5	7	3
18	F-box/SPRY domain-containing protein 1	0	1	0	2	2	1	0	0	0
19	Zinc-alpha-2-glycoprotein	6	3	6	2	3	2	3	4	1
20	Hemoglobin subunit beta	7	9	9	0	0	0	2	0	0
21	Alpha-1-antichymotrypsin His-Pro-less	5	2	3	10	11	1	4	3	3
22	Retinol-binding protein 3	5	3	8	1	1	0	2	1	1
23	Complement C3	1	0	0	4	5	1	2	1	3
24	Ig gamma-2 chain C region	13	11	16	0	0	0	0	0	0
25	Ig gamma-4 chain C region	8	10	13	0	0	0	0	2	0
26	Apolipoprotein A-II	3	2	2	2	4	2	0	0	2
27	Haptoglobin	1	1	2	1	2	1	3	1	1
28	Alpha-2-HS-glycoprotein chain A	0	1	1	3	1	4	0	0	0
29	Antithrombin-III	0	0	0	2	1	3	0	0	0
30	Ig gamma-3 chain C region	12	11	6	0	0	0	0	0	0
31	Gelsolin	0	0	3	0	0	0	0	0	0
32	Protein shisa-7	0	0	0	0	2	0	0	0	0
33	Dickkopf-related protein 3	0	3	0	2	1	0	0	0	1

**Table 4:** List of proteins found to be reduced significantly by ANOVA in the patient samples compared to the control.

**Identified Proteins**	**Accession Number**	**ANOVA Test (P-Value)**
Ig gamma-1 chain C region	IGHG1_HUMAN	0.022
Retinol-binding protein 3	RET3_HUMAN	0.016
Serotransferrin	TRFE_HUMAN	0.011
Prostaglandin-H2 D-isomerase	PTGDS_HUMAN	0.0028
Ig gamma-3 chain C region	IGHG3_HUMAN	0.0027
Ceruloplasmin	CERU_HUMAN	0.0014
Pigment epithelium-derived factor	PEDF_HUMAN	0.00053
Hemoglobin subunit beta	HBB_HUMAN	0.000059
Ig gamma-4 chain C region	IGHG4_HUMAN	0.00004
Ig gamma-2 chain C region	IGHG2_HUMAN	0.0000056
